# Decreased Intraoperative Renal Tissue Oxygenation after Cardiopulmonary Bypass Predicts Cardiac Surgery-Associated Acute Kidney Injury in Neonates

**DOI:** 10.3390/children11030315

**Published:** 2024-03-07

**Authors:** Paige E. Condit, Daniel P. Gorski, Michael R. Lasarev, Awni M. Al-Subu, Matthew W. Harer

**Affiliations:** 1 Division of Neonatology, Department of Pediatrics, School of Medicine and Public Health, University of Wisconsin, Madison, WI 53705, USA; dpgorski@wisc.edu (D.P.G.); mwharer@wisc.edu (M.W.H.); 2 Department of Biostatistics and Medical Informatics, School of Medicine and Public Health, University of Wisconsin, Madison, WI 53705, USA; lasarev@biostat.wisc.edu; 3 Division of Critical Care, Department of Pediatrics, School of Medicine and Public Health, University of Wisconsin, Madison, WI 53705, USA; al-subu@pediatrics.wisc.edu

**Keywords:** NIRS, congenital heart disease, noninvasive

## Abstract

(1) Background: Near-infrared spectroscopy (NIRS) is a noninvasive tool frequently used during cardiac surgery and postoperatively in the cardiac intensive care unit to monitor regional tissue oxygen saturation. A relationship between trends of intraoperative renal oxygenation and the risk of developing cardiac surgery-associated acute kidney injury (AKI) post-operatively has not yet been established in the neonatal population. The objective of this study is to evaluate the relationship of cerebral and renal oxygenation during cardiopulmonary bypass with cardiac surgery-associated AKI in the first 72 h post-operation in neonates < 30 days of age. (2) Methods: A prospective cohort study at a tertiary care children’s hospital was performed. Renal and cerebral oxygenation measured were collected intraoperatively from neonates < 30 days of age who underwent cardiopulmonary bypass for the correction of congenital heart disease. AKI was defined accordance with the Kidney Disease: Improving Global Outcomes criteria modified for neonates. Variables were compared between groups. (3) Results: 32 neonates with 35 cardiopulmonary bypass cases were included. AKI was diagnosed in 60% of cases. Intra-operative renal oxygenation, both on- and off-bypass, did not differ among the three AKI groups (*p* > 0.19). Renal oxygenation after coming off, but not during, cardiopulmonary bypass steadily decreased with increasing levels of AKI (Jonckheere’s test, one-sided *p* = 0.024). (4) Conclusions: Renal oxygenation decreased in proportion to AKI severity after coming off, but not during, cardiopulmonary bypass.

## 1. Introduction

Acute kidney injury (AKI) is common in neonates and is associated with longer hospitalization, increased morbidity and mortality, and the development of chronic kidney disease [1,2,3]. Undergoing cardiopulmonary bypass during the surgical correction of congenital heart disease frequently leads to cardiac surgery-associated AKI [4], particularly in neonates. The etiology of this process is multifactorial, and includes hypoperfusion/reperfusion injury, coagulopathy, oxidative stress, and inflammation [5].

The most accepted criteria for the diagnosis of AKI in this population is the Kidney Disease: Improving Global Outcomes (KDIGO) definition modified for neonates [6]. While changes in serum creatinine and urine output are used by clinicians to define and diagnose AKI, these are lagging markers that present only after damage to renal tissue has occurred [7]. Additionally, neonates who undergo surgery in the first week of life often have baseline creatinine levels reflective of maternal levels, which may lead to missed diagnoses of cardiac surgery-associated AKI [8]. Recent studies have evaluated urinary biomarkers, such as neutrophil gelatinase-associated lipocalin and cystatin C, as earlier markers of AKI; however, they have not shown consistent performance, particularly in neonates undergoing surgical repair for congenital heart disease [9,10].

Near-infrared spectroscopy (NIRS) monitoring is frequently used during cardiopulmonary bypass to monitor regional tissue oxygen saturation (rSO_2_) [11], providing an estimate of regional tissue oxygen utilization. NIRS monitoring can provide insights regarding tissue perfusion as well as the ability of the tissue to extract and utilize oxygen, and has been shown to correlate with lactate levels in children after undergoing cardiac surgery [12]. Studies have shown that observed changes in intra-operative renal rSO_2_ values are associated with cardiac surgery-associated AKI [11,13]. The ability to detect cardiac surgery-associated AKI prior to changes in serum creatinine and urine output may provide clinicians with the ability to prevent worsening AKI. The objective of this study was to evaluate the relationship between intra-operative renal rSO_2_ values and the post-operative development of cardiac surgery-associated AKI in neonates.

## 2. Materials and Methods

This prospective cohort study was conducted at American Family Children’s Hospital (Madison, WI, USA) between January 2020 and October 2021. The Health Sciences Institutional Review Board at the University of Wisconsin–Madison approved this study (2019-1504) and determined that consent was not required, as the monitoring performed in this study was determined to be standard-of-care. Neonates < 30 days of age who underwent cardiopulmonary bypass for the correction of congenital heart disease were included. A major renal anomaly was defined as any congenital anomaly of the kidney identified on prenatal ultrasound. AKI was defined in accordance with the modified neonatal KDIGO definition including urine output. Severe AKI was defined as stage 2 or 3 based on previous neonatal AKI publications that have grouped AKI severity [14]. Serial creatinine values and urine output were collected for 72 h post-operatively (post-operative day 1–3) for each patient.

Cerebral and renal rSO_2_ values were recorded every 6 s with INVOS 5100C NIRS monitors (Medtronic, Minneapolis, MN, USA) and neonatal sensors. The cerebral sensor was placed midline on the infant’s forehead, and the renal sensor was placed to the right or left of the spine at the T10 to L2 vertebral level prior to starting the operation on the neonate. Baseline data were collected for each patient in the hour prior to cardiopulmonary bypass. The durations each patient was on cardiopulmonary bypass were analyzed separately. The time points after each patient came off cardiopulmonary bypass for the final time were also analyzed separately.

Demographic data, including gestational age at birth, birth weight, sex, and day of life at time of surgery, were collected. The Society of Thoracic Surgeons-European Association for Cardio-Thoracic Surgery category (STAT) and Risk Adjustment for Congenital Heart Surgery scores (RACHS-1) were obtained for each case. The total time spent on cardiopulmonary bypass for each case was also recorded.

The geometric mean was used to summarize each patient’s intra-operative renal %rSO_2_ on-bypass, off-bypass, and after coming off bypass for the last time. The geometric mean was selected to summarize the intra-operative data due to the presence of ratios used in the analysis (renal:cerebral NIRS and on:off bypass for each NIRS component) and the rule that the geometric mean for a collection of ratios equals the ratio of geometric means computed from the constituent parts. Summary values of the geometric mean were computed for cerebral and renal NIRS as well as the ratio (renal:cerebral). The median and interquartile range (IQR) were used to describe these summary values according to the stage of AKI with the Kruskal–Wallis test being used to test for associations between AKI stage and summary NIRS response during the final bypass cycle and the subsequent hour off bypass. Jonckheere’s test was used to determine whether the summary NIRS response followed an increasing or decreasing trend as a function of AKI severity.

## 3. Results

In total, 32 neonates underwent repair of congenital heart disease during this study period, accounting for 35 separate surgeries on cardiopulmonary bypass as three neonates returned to the operating room a second time during the first 30 days of life: one patient returned to the operating room for a revision of their right ventricle to the pulmonary artery (Sano) conduit due to severe proximal stenosis, and one patient required Damus–Kaye–Stansel (DKS) revision with a patch of the orifice of the right coronary artery, and the last patient required a second operation for a right-ventricular free wall pseudoaneurysm. None of the infants had a pre-operative diagnosis of major renal anomaly. There were no statistically significant differences in gestational age, birth weight, sex, or mortality rates when comparing AKI outcome groups (Table 1—Demographics). Overall mortality in this cohort was 19% (6 of 32). One patient required kidney replacement therapy. Neonates had multiple different types of cardiac repair, with the most common one being a stage 1 Norwood procedure (Table 2).

AKI was diagnosed in 60% (21 of 35) of cases, with 29% (10 of 35) having severe AKI (stage 2/3) (Table 3). Of the 21 AKI cases, 29% (6 of 21) were diagnosed due to an increase in serum creatinine, 33% (7 of 21) were diagnosed due to a decrease in urine output, and 38% (8 of 21) met both diagnostic criteria. Of the 10 cases of severe AKI (stage 2/3), only two of them were diagnosed due to an increase in serum creatinine alone. The pre-op creatinine and peak 72 h post-op creatinine values did not differ significantly between groups. Additionally, there was no statistically significant association between total time on cardiopulmonary bypass and the development of AKI. There was no statistically significant correlation between Risk Adjustment for Congenital Heart Surgery (RACHS-1) score, The Society of Thoracic Surgeons-European Association for Cardio-Thoracic Surgery (STAT) score, and AKI diagnosis.

**Figure 1:**
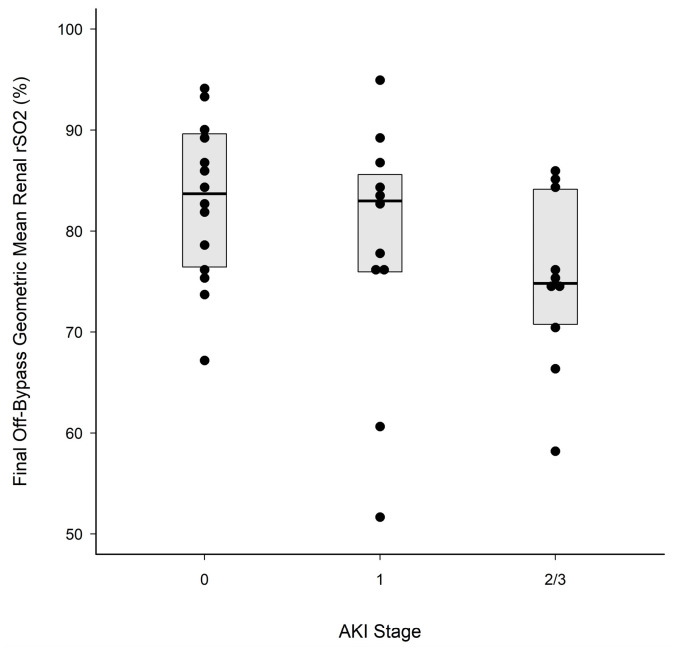
Geometric mean off-bypass. The central solid line in each box marks the median renal %rSO_2_ after coming off bypass for the final time; upper and lower box edges mark the 75th and 25th percentiles, respectively. Solid black circles are individual measurements. Renal rSO_2_ (median [IQR]) after coming off the last cardiopulmonary bypass cycle steadily decreased with increasing levels of AKI. Stage 0 = 83.5 (77.0, 89.1); stage 1 = 81.2 (71.1, 86.7); stage 2/3 = 75.4 (69.5, 81.9); Jonckheere’s one-sided test, *p* = 0.024.

Intra-operative cerebral and renal rSO_2_, both on- and off-bypass, did not differ among the three AKI groups (*p* > 0.19, Table 4) when analyzed as unordered groups without considering the AKI stage. Additionally, the ratio of renal to cerebral rSO_2_ on- and off-bypass was not different between groups. However, when analyzing the data with a consideration of AKI severity (0 < 1 < {2/3}), renal rSO_2_ after coming off the last cardiopulmonary bypass cycle steadily decreased with an increasing level of AKI (stage 0 = 83.5 (77.0, 89.1); stage 1 = 81.2 (71.1, 86.7); stage 2/3 = 75.4 (69.5, 81.9); Jonckheere’s one-sided test, *p* = 0.024) (Figure 1).

## 4. Discussion

Neonates who undergo cardiopulmonary bypass during the surgical correction of congenital heart disease are at significant risk for developing cardiac surgery-associated AKI [4], which leads to an increase in morbidity and mortality [15,16]. In this prospective observational cohort study, we found that renal rSO_2_ did not differ between AKI groups during cardiopulmonary bypass but decreased significantly in proportion to AKI severity during the 60 min after completing the final cardiopulmonary bypass cycle. A separate analysis of the renal and cerebral rSO_2_ data during complete intra-operative monitoring failed to demonstrate statistically significant differences between the AKI outcome groups. Our observed cardiac surgery-associated AKI incidence of 60% was consistent with that observed in a recent large multicenter retrospective study [17]. Given the high rates of AKI in this population, and our significant findings, further studies are required to prospectively determine if treatment after cardiopulmonary bypass can be altered based on renal rSO_2_ to prevent or reduce the severity of AKI.

Our findings of no differences during cardiopulmonary bypass but differences after the last cycle of cardiopulmonary bypass have several possible explanations. First, during cardiopulmonary bypass, the anesthesia, perfusion, and surgeon teams focus on maintaining oxygenation, and if significant hypoxia occurs, take corrective actions—thus explaining our lack of significant differences during bypass. However, after coming off cardiopulmonary bypass, less interventions are available to correct hypoxia, and there is a higher reliance on intrinsic cardiac function to maintain perfusion and normoxia. Thus, one potential explanation for the lower renal oxygenation immediately after cardiac repair is that patients showing this have worse intrinsic cardiac function. Another explanation could be that the inflammatory response after coming off bypass is higher in patients with lower renal oxygenation, and this contributes to the increased risk of AKI [18].

NIRS studies often incorporate complex statistical analysis to retroactively determine the risk of cardiac surgery-associated AKI after the completion of cardiopulmonary bypass. This is often not practical for clinical decision making in real time. Opportunities to mitigate the risk of cardiac surgery-associated AKI and optimize fluid removal should be considered in real time while in the operating room. One such intervention to consider prior to leaving the operating room is to prophylactically place a peritoneal catheter [19]. One of the strengths of our study is that we were able to analyze a specific period towards the end of the surgical procedure, which did not require complex statistical calculations. Given our findings, a renal rSO_2_ value of less than 70 in the 1 h post-cardiopulmonary bypass would suggest a higher risk of stage 2 or 3 AKI, in which case the placement of a peritoneal catheter may be an appropriate consideration. Another potential strategy that would need a prospective clinical study is the use of a methylxanthine medication, like theophylline or caffeine, when leaving the operating room. In neonates with hypoxic ischemic encephalopathy, a single dose of theophylline in the first six hours of birth reduces the rate of AKI [20]. In preterm neonates, caffeine has been shown to improve renal oxygenation, and those on caffeine have reduced rates of AKI [21,22]. Finally, and most importantly, children with low rSO_2_ in the 1 h post-cardiopulmonary bypass may require additional attention in avoiding additional hits to the kidneys, including the administration of nephrotoxic drugs, and ensuring an appropriate level of hydration, optimal end organ perfusion, and optimal oxygen delivery.

One limitation of our study is the low number of neonates who met the study’s inclusion criteria, which reduced the statistical power to detect differences among AKI groups. Additionally, significant variability can occur during the post-operative period, which may impact the development of AKI in the first 72 h, including low cardiac output syndrome, the use of nephrotoxic medications, fluid management, and diuretic use. These potentially confounding variables were not accounted for in this study but are unlikely to cause intra-operative AKI. In addition, during the study period, our observed mortality was higher than expected, which could reflect multi-organ dysfunction developing later in patients’ course and not detected via intraoperative NIRS monitoring.

In conclusion, our study demonstrates that the analysis of renal rSO_2_ after the completion of bypass may provide an earlier indication of which neonates are at greatest risk of developing high-grade AKI. Future larger multicenter prospective studies using urinary samples to analyze urinary biomarkers indicative of kidney injury together with renal NIRS values may be more predictive of cardiac surgery-associated AKI. An AKI risk score when leaving the operating room that includes renal oxygenation could lead to earlier and improved interventions that prevent significant AKI.

## Figures and Tables

**Table 1:** Demographics (per patient).

	No AKI (n = 13)	Stage 1 (n = 10)	Stage 2/3 (n = 9)	*p*-Value
Gestational age at birth (weeks)	39.0 (38.0–39.4)	38.6 (38.1–39.1)	39.0 (38.6–39.1)	0.536
Birth weight (kg)	3.27 (2.77–3.52)	3.40 (3.04–3.59)	3.03 (2.75–3.44)	0.540
Mortality	1 (8%)	2 (20%)	3 (33%)	0.321
Female	7 (54%)	4 (40%)	4 (44%)	0.90

**Table 2:** Type of cardiac surgery.

Surgical Repair	Number of Infants
Stage 1 Norwood	12
Aortic arch repair	7
Arterial switch	4
Total anomalous pulmonary venous return repair	3
Pulmonary artery banding	2
Truncus arteriosus repair	2
Revision of right ventricle to pulmonary artery (Sano) conduit	1
Central Gore-Tex shunt placement	1
Closure of right ventricular free wall pseudoaneurysm	1
Double outlet right ventricle repair	1
Revision of DKS anastomosis and left pulmonary artery plasty	1

**Table 3:** Demographics (per case).

	No AKI (n = 14)	Stage 1 (n = 11)	Stage 2/3 (n = 10)	*p*-Value
Pre-operative creatinine (μmol/L)	51.3 (39.8–0.64.6)	45.1 (25.6–56.6)	41.6 (23.9–89.3)	0.24
Peak 72 h post-operative creatinine (μmol/L)	62.8 (51.3–77.8)	73.4 (55.7–82.2)	78.7 (61.9–114.1)	0.343
Lowest 72 h post-operative urine output (ml/kg/h)	0.91 (0.64–1.30)	0.43 (0.36–0.63)	0.33 (0.19–0.68)	<0.001
Cardiopulmonary bypass time (hours)	2.50 (1.97–3.41)	2.90 (2.53–3.12)	2.73 (1.88–4.54)	0.605
RACHS-1 ^1^ Score > 3	10 (71%)	7 (64%)	7 (70%)	1
STAT ^2^ Category > 3	11 (79%)	9 (82%)	9 (90%)	0.861

^1^ RACHS-1—Risk Adjustment for Congenital Heart Surgery score; ^2^ STAT score—The Society of Thoracic Surgeons-European Association for Cardio-Thoracic Surgery.

**Table 4:** Intra-operative renal and cerebral rSO_2_, on- and off-bypass, stratified by AKI stage.

	No AKI (n = 14)	Stage 1 (n = 11)	Stage 2/3 (n = 10)	*p*-Value
Renal (on cardiopulmonary bypass)	69.1 (62.5, 76.5)	64.4 (55.4, 74.9)	74.7 (68.7, 81.2)	0.185
Renal (off cardiopulmonary bypass)	75.3 (70.0, 81.0)	70.9 (62.9, 79.9)	72.1 (68.3, 76.1)	0.556
Cerebral (on cardiopulmonary bypass)	70.9 (65.0, 77.4)	72.2 (65.9, 79.2)	72.3 (64.7, 80.9)	0.944
Cerebral (off cardiopulmonary bypass)	65.2 (59.0, 72.2)	65.7 (58.5, 73.7)	63.1 (54.9, 72.5)	0.894
Renal/Cerebral (on cardiopulmonary bypass)	0.97 (0.84, 1.13)	0.89 (0.75, 1.06)	1.03 (0.89, 1.20)	0.428
Renal/Cerebral (off cardiopulmonary bypass)	1.15 (1.05, 1.26)	1.08 (0.94, 1.23)	1.14 (1.03, 1.27)	0.698

## Data Availability

The data presented in this study are available on request from the corresponding author. The data are not publicly available due to privacy restrictions.
